# From diagnostics to education: Multi-domain evaluation of LLM chatbots in neurology

**DOI:** 10.1016/j.jtumed.2025.11.004

**Published:** 2025-12-26

**Authors:** Gopi Battineni, Nalini Chintalapudi, Venkata R. Dhulipalla, Francesco Amenta

**Affiliations:** a Biocomputing Developmental Systems Research Group, Department of Computer Science andInformation Systems, University of Limerick, Limerick, Ireland; b Research Centre of ECE department, Siddhartha Academy of Higher Education, Vijayawada, India; c Research Department, International Radiomedical Centre (C.I.R.M.), Rome, Italy

**Keywords:** طب الأعصاب, النموذج اللغوي الضخم, الفحوصات التخصصية, الاعتبارات الأخلاقية, ChatGPT4, Ethical concerns, Large language model, Neurology, Specialist examinations

## Abstract

**Objectives:**

The development of large language models (LLMs) has shown promising results in enhancing research processes, data analysis, and communication in various domains of neurology. In this work, we systematically review and synthesize current evidence on the applications of LLMs in the assessment, diagnosis, and monitoring of neurological disorders.

**Methods:**

Three databases, namely PubMed, Scopus, and Web of Science, were considered for document search. Article selection was according to PRISMA guidelines, and Newcastle–Ottawa Scale (NOS) was used to assess the article quality based on relevance, quality, and applicability.

**Results:**

Nine studies were included in the final analysis. Based on the findings, LLMs have been utilized in diverse areas of neuroscience including hypothesis generation, clinical decision support, and cognitive modeling. LLMs can process large datasets, identify trends, and support personalized medicine. However, challenges such as interpretability, ethical considerations, and domain-specific training remain critical.

**Conclusions:**

By facilitating workflows and uncovering new insights, LLMs can revolutionize different domains of neurology. Nevertheless, further research on their reliability, ethical implications, and adaptation to the unique demands of neuroscience is needed.

## الملخص

**أهداف البحث:**

أظهرت النماذج اللغوية الضخمة تقدّمًا ملحوظًا في دعم عمليات البحث العلمي، وتحليل البيانات، وتعزيز الاتصال في مختلف فروع علوم الأعصاب. تهدف هذه الدراسة إلى إجراء مراجعة منهجية وتجميع الأدلة الحالية حول تطبيقات النماذج اللغوية الضخمة في تقييم واضطراب وتشخيص ومتابعة الاضطرابات العصبية.

**طرق البحث:**

تم البحث في ثلاثة قواعد بيانات هي: قاعدة بيانات العلوم الطبية الحيوية، وقاعدة بيانات سكوبس، وقاعدة بيانات العلوم. جرى اختيار الدراسات وفقًا لإرشادات "بيزما"، كما تم استخدام مقياس "نيوكاسل–أوتاوا" لتقييم جودة الدراسات من حيث الصلة والمنهجية وقابلية التطبيق.

**النتائج:**

شملت المراجعة تسع دراسات في التحليل النهائي. تشير النتائج إلى توظيف النماذج اللغوية الضخمة في مجالات متعددة من علوم الأعصاب، بما في ذلك توليد الفرضيات، ودعم اتخاذ القرار السريري، والنمذجة الإدراكية. يمكن لهذه النماذج معالجة مجموعات ضخمة من البيانات، واستخلاص الأنماط، ودعم الطب الشخصي. ومع ذلك، ما تزال تحديات مثل قابلية التفسير، والاعتبارات الأخلاقية، والحاجة إلى تدريب متخصص تمثّل نقاطًا حرجة.

**الاستنتاجات:**

من خلال تسهيل سير العمل وكشف رؤى جديدة، تمتلك النماذج اللغوية الضخمة القدرة على إحداث تحول جذري في مختلف مجالات علوم الأعصاب. ومع ذلك، تظل الحاجة قائمة لمزيد من الدراسات التي تبحث في موثوقيتها، وآثارها الأخلاقية، ومدى تكيفها مع المتطلبات الخاصة لعلم الأعصاب.

## Introduction

Accurate and timely diagnosis of diseases is crucial in the medical field for effective treatment and patient care.^1^ Large language models (LLMs) can process and interpret unstructured medical data due to their natural language processing capabilities.^2^^,^^3^ Continuous learning and improvement over time is one of the main advantages of LLMs.^4^^,^^5^ This involves comprehending and summarizing patient symptoms, medical histories, and other relevant information from electronic health records.^6^^,^^7^ LLMs have the potential to support earlier detection and optimize disease classification, helping neurologists make more informed diagnostic decisions. It is believed that they can be integrated into existing clinical workflows to support doctors in this process.^8^

It is believed that LLMs can provide decision support by suggesting potential diagnoses based on patient data analyses.^9^^,^^10^ Integrating these models can reduce the cognitive load of clinicians and improve diagnostic process efficiency. Complex medical data including patient records, clinical notes, and research articles are analyzed by LLMs to find patterns and correlations that may not be immediately apparent to human clinicians. The training of LLM models on large amounts of text data allows them to understand and generate texts that resemble human language, which is essential in the diagnosis of neurodegenerative disorders.^11^

The huge amount of data that LLMs are trained on can be utilized to create personalized medicine approaches.^4^ Their assistance in identifying specific biomarkers and genetic factors associated with neurological disorders/neurodegenerative diseases can result in more tailored and effective treatment plans for individual patients. Early diagnosis can assist patients with neurological disorders in modifying their lifestyle to slow down disease progression. Due to recent advancements in artificial intelligence (AI) and natural language processing models such as ChatGPT, medical diagnostics show significant potential in the healthcare domain.^12^^,^^13^

The increased availability of neurological data and refinement of these models will increase their diagnostic accuracy and utility in clinical settings. However, researchers argue that the use of LLMs in medical diagnostics also presents challenges that include ensuring data privacy and security, addressing biases in the training data, and obtaining regulatory approval for clinical use.^14^ The need for clinicians to understand the rationale behind the recommendations of LLMs means that the interpretability of these models remains a critical area of research.

A recent review explored LLM-based diagnostic methods, covering disease types, clinical data, techniques, and evaluation while highlighting research gaps and future directions.^15^ Another study examined how LLMs are revolutionizing digital diagnostics, and the rapid development is revolutionizing digital diagnostics in healthcare.^13^ This study evaluated their diagnostic abilities, highlighting ChatGPT4 high accuracy, Gemini's precision in high-risk diagnoses, and ChatGPT-3.5's utility and emphasizing the importance of patient privacy and ethical considerations in applying LLMs in medical settings. These concepts were complemented by Yu et al.,^16^ who discusses the importance of LLMs in biomedical and health informatics, providing new ways to analyze data, treat patients, and conduct research.

Keeping LLMs updated with the latest medical knowledge and practices is essential and challenging. This should be highest priority while transforming LLM in the diagnosis of neurological/neurogenerative disorders; however, a comprehensive understanding of their application and evaluation is still needed. It is also important to ensure the privacy of patients, and rigorous validation is needed to ensure the reliability and accuracy of LLMs in neuroscience. Effective integration of LLM requires collaboration among neuroscience, AI, and clinical practice, which can be challenging to manage.^17^^,^^18^ The rapid evolution of the field makes it challenging to keep LLMs up-to-date with the latest neuroscientific knowledge and practices, which is essential.^18^

There is limited empirical evidence that LLMs are effective in assessment areas of neurology. Models such as ChatGPT (different versions), Bard, and Claude may enhance diagnostic support in neurodegenerative diseases. These are which diagnosed through clinical history, linguistic analysis, and cognitive testing.^19^ The LLM chatbots could improve decision-making and streamline workflows in these areas. It is hard to tell which models offer the most comprehensive capabilities while maintaining efficiency, accuracy, and usability. Neither clinical nor research contexts are exempt from this requirement. A comparative evaluation of Chatbots in the context of neurodegenerative disorder assessment addresses these gaps, identifying their strengths and limitations, exploring ethical and technical issues, and proposing future directions for integrating LLMs into neurology in a safe, effective, and responsible way.

## Materials and Methods

### Document search

A literature search was conducted using three major databases, PubMed (Medline), Scopus, and Web of Science (WoS). Search strategies were created to locate important literature on LLM applications, challenges, and prospects in neuroscience. Synonyms, alternative terms, and substitutes for key terminologies were coupled with Boolean operators namely “LLM applications” OR “LLM in neuroscience” OR “LLM AND brain function analysis” OR “cognitive modeling with LLM” OR “LLM AND neuroimaging” OR “chatbot applications in mental health” AND “transformative impacts of LLM on neuroscience research.” The search for documents was carried out according to the guidelines provided by Preferred Reporting Items for Systematic Reviews and Meta-Analyses (PRISMA) 2020.^20^ By reviewing abstracts and drafting a list of relevant articles, the authors conducted an initial appraisal independently. To reach a consensus on the final list of eligible articles, a consensus was reached by reviewing discrepancies in selected search terms and discussing different interpretations. After the articles were approved for inclusion, a thorough review was conducted to obtain relevant data supporting the research objectives. An agreement was reached by performing the evaluation process independently and comparing opinions.

### Inclusion and exclusion criteria

This study analyzed full-text papers that were published in English in peer-reviewed journals only. The papers selected were mainly focused on how LLM can be used in neuroscience research, emphasizing advancements in neuroimaging, cognitive modeling, disease diagnosis, and brain–computer interfaces. The inclusion of studies was contingent upon their involvement in LLM applications in mental health, brain function analysis, or transformative impacts on neuroscience. Eligible papers were required to have a title or abstract containing at least one relevant keyword associated with LLM integration in neuroscience. Papers included for review were limited to years from 2018 to 2025. In 2018, the emergency of generative pretrained transformers began after LLMs’ advanced and practical applications decreased the relevance of previous studies.^21^ All papers published in languages other than English, or outside the time frame mentioned, were excluded. We also excluded all studies that did not specifically focus on LLM applications in neuroscience or that did not pertain to clinical diagnosis, cognitive modeling, or neuroimaging. A further exclusion was made of research that relied on imaging modalities rather than neuroimaging linked to LLMs. To maintain a focus on original research, review articles, conference proceedings, and gray literature were also omitted.

### Paper screening and validation

Several stages were taken in the selection process to ensure that high-quality and relevant papers were selected. Following a predetermined search strategy, the results of the systematic search were meticulously documented in a spreadsheet. To identify potentially relevant publications, the titles and abstracts of the identified papers were distributed equally among the authors for initial screening. To ensure a structured and rigorous approach to article selection was done by the PRISMA guidelines with three stages, namely Identification, Screening, and Inclusion.^22^ The selected papers were then subjected to a comprehensive review based on predefined inclusion and exclusion criteria. The quality of the included studies was assessed using the Newcastle–Ottawa Scale (NOS), a structured framework designed to evaluate bias risk in three primary domains: selection, comparability, and outcome.^23^ Each study received a score reflecting its performance across these domains, with scores ranging from very poor to excellent. A score of 7 or above indicated high quality, aiding researchers in identifying trustworthy studies. A spreadsheet of NOS outcomes for selected works was provided. To ensure impartial evaluation, two authors (GB and NC) independently assessed each study and compared their scores. Disagreements were discussed to settle divergent viewpoints, and additional consultations were undertaken with a third author (VRD) and neurological specialist (FA) to address any unresolved discrepancies. This meticulous quality assessment process ensured that only robust and reliable studies were included in the synthesis and analysis of LLM applications in neurology.

## Results

### Article selection outcomes

**Figure 1:**
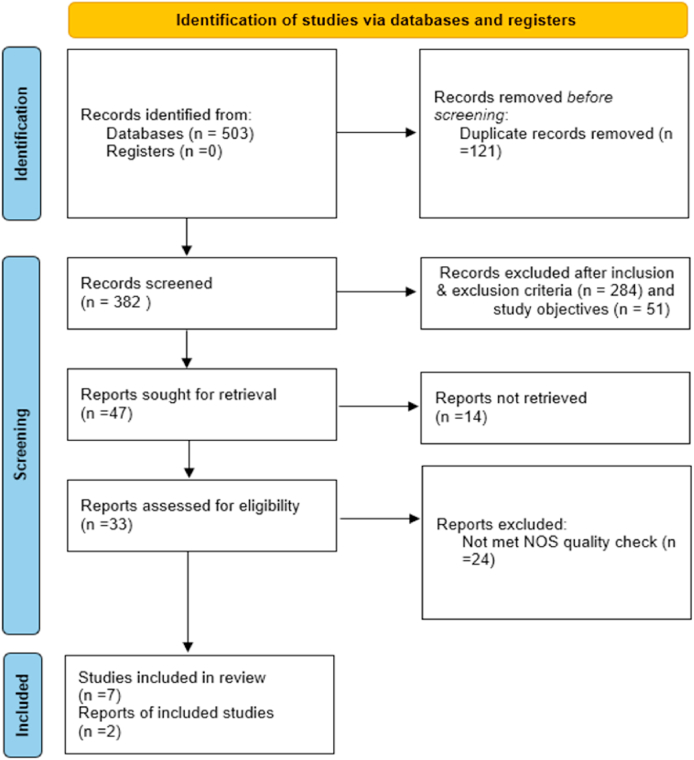
Article screening flow chart based on PRISMA 2020 guidelines.

The flow chart in Figure 1 shows the process of article selection for the review. The literature search identified 503 records from three major scientific databases using the specified search strategy: PubMed (n = 416), Scopus (n = 58), and WoS (n = 29). After reviewing titles and abstracts in the initial phase, 121 papers were eliminated due to duplication, and the remaining papers were accepted for further screening. In a follow-up, we excluded 335 works because they did not align with the inclusion and exclusion criteria (n = 284) and study objectives (n = 51). Fourteen records were not considered because the full text was not retrieved, and 33 papers were transferred to quality check to ensure they followed the NOS guidelines. After conducting quality checks, nine studies were found to have met the criteria and were included in the final analysis.

### Study characteristics

The characteristics of each study were structured in tabular format to facilitate the synthesis by extracting key information from each study. In addition to data on the design of the study, datasets, performance metrics, model validation techniques, and applications of LLMs in neuroscience, the extracted data are provided in Table 1. To identify common themes, patterns, and trends throughout the studies, a summary of the main findings was compiled. The contributions and limitations of LLM in neuroscience research were examined, including applications such as neuroimaging, cognitive modeling, and disease diagnosis. By doing this, we made sure that the analysis was comprehensive and focused on the research objectives.

**Table 1:** Study characteristics.

Author	Place of study	Study type	Trained data	LLM Models	Metrics	Major findings	LLM potentiality
Kanzawa et al., 2024	Japan	Retrospective	759 brain MRI reports	Optimized BERT	Accuracy, sensitivity, and specificity	LLM accuracy, sensitivity, and specificity were not significantly different from human readers (p = 0.371).	LLM cannot fully replace doctors because complex cases still require human expertise
Schubert, Wick, and Venkataramani 2023a	Germany	Cross-sectional	An exam bank modeled after neurology board-style questions	ChatGPT3.5 (LLM 1) & ChatGPT4 (LLM 2)	Comparing lower-order and higher-order questions, and overall percentage scores of LLMs	LLM2 outperformed LLM1 and the mean human score, achieving 85.0 % correct answers compared to 73.8 % for humans	Clinical neurology applications for LLM show significant potential
Chen et al., 2023	USA	Proof-of-concept	Using reinforcement learning from human feedback with medical data as input	Microsoft bing chat coupled with ChatGPT	Average error rate	With ChatGPT, efficiency, and accuracy are expected to significantly improve in medicine	The ChatGPT demonstrated limited accuracy and error susceptibility when evaluated with established neurological assessment scales
Williams et al., 2024	UK	Prospective comparative	Neurosurgical interview questions, scored by neurosurgical consultants	ChatGPT3.5	ChatGPT and human interview scores were compared	ChatGPT did not outperform human participants in training positions	While ChatGPT underperformed, the study suggests future collaboration between humans and AI could be efficient in neuroscience
Giannos 2023	UK	Comparative evaluation	69 questions from the SCE neurology web questions bank	ChatGPT3.5 legacy, ChatGPT 3.5 default & ChatGPT4	Accuracy	In the 2022 SCE neurology exam, ChatGPT4 achieved a passing rate of 58 %, outperforming its predecessors	ChatGPT4 has the potential to improve the quality and relevance of specialized medical education and practice
Cano-Besquet et al., 2024	USA	Retrospective cohort	Patient data from the Epic EHR system	ChatGPT4	Accuracy, comprehensiveness scores, success rates	There was no statistically significant difference between ChatGPT4 and consultant neurologists in diagnostic accuracy	ChatGPT4 could serve as a valuable diagnostic tool in inpatient neurology, providing comprehensive and accurate initial diagnoses
Shojaee-Mend et al., 2024	Iran	Comparative evaluation	20 neurophysiology questions	ChatGPT, Google's bard, and Anthropic's claude	Mean score	LLMs demonstrated good performance with a mean score of 3.87 out of 5. There was no significant difference between languages.	LLMs showed proficiency in neurophysiology
Hewitt et al., 2024a	Germany	Comparative evaluation	30 challenging neuropathology cases, each presenting a complex mix of morphological and genetic information relevant to the diagnosis.	ChatGPT-4o, claude-3.5-sonnet, and Llama3	Accuracy	LLMs equipped with RAG can effectively diagnose complex neuro-oncology cases.	RAG-equipped LLMs can effectively diagnose neuro-oncology
Ros-Arlanzón and Perez-Sempere 2024	Spain	Comparative analysis	2022 neurology specialist examination results from 120 neurologists	ChatGPT3.5 & ChatGPT4	Median scores	ChatGPT3.5 ranked 116th out of 122, with 54.5 % correct answers and ChatGPT4 ranked 17th, with 81.8 % correct answers	ChatGPT4 showed a high level of proficiency in answering neurology specialist examination questions

#### Study design

The types of studies that were adopted are listed in this section, which provides information about the prevalence and focus of each study. Comparative analysis is the primary method used in this review with four studies utilizing it.^28^^,^^30^, ^31^, ^32^ The reason for this preference is that it is aligned with evaluating outcomes when using LLM techniques. The retrospective approach is the second most utilized, represented in two studies.^24^^,^^29^ Compared to prospective designs, conducting studies with existing datasets is easier, but requires longer timelines. Other categories such as cross-sectional, proof-of-concept, and prospective comparative were each used in only one study, indicating heterogeneity in these methodologies.^25^, ^26^, ^27^ The resource-intensive nature of prospective comparative approaches could be the cause of their underrepresentation.

#### Adopted LLM models

**Figure 2:**
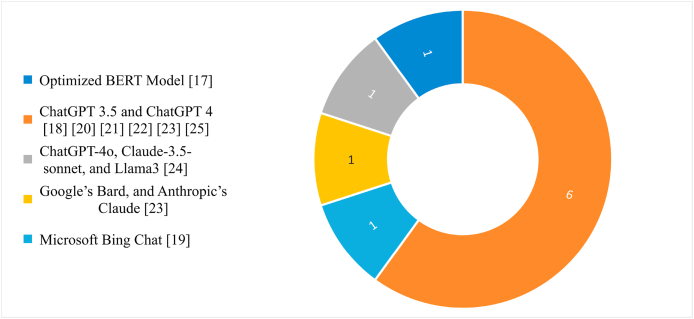
Study count based on the incorporation of different LLM models.

Figure 2 illustrates the distribution of included studies based on the incorporation of different LLM models. The studies involved attempted to use of variety of different LLM models reflecting diversity and progression. Three major LLMs, namely OpenAI models such as ChatGPT, Google models such as Bard, and Anthropic models such as Claude-3.5-sonnet were used. The widespread adoption of ChatGPT 3.5 and 4 is demonstrated by the use of a combination in most studies.^25^^,^^27^, ^28^, ^29^, ^30^^,^^32^ In various studies, optimized and legacy versions, such as ChatGPT-4o and ChatGPT 3.5 Legacy, were found.^28^^,^^31^ A different group of papers included broader comparisons that involved models such as Bard, Claude, Llama3, and optimized versions, and highlighted the effort to benchmark across providers.^30^^,^^31^ One study stands out for its focus on a specialized pretrained Japanese BERT model.^24^ The LLM landscape in neuroscience research is dominated by OpenAI, Google, and Anthropic, which is due to their leadership in providing state-of-the-art NLP models. Although other studies have utilized prominent models, emerging alternatives like Llama3 should be investigated more extensively for specific neurological applications.

#### Model training and data source evaluation

In this review, a variety of neurology-related datasets have been used to train, fine-tune, or evaluate LLMs, spanning both academic and clinical settings. In addition to 759 brain MRI results,^24^ which provided real-world radiological narratives for neuroimaging interpretation, there were also patient data from the Epic Electronic Health Record, which provided structured and unstructured information for diagnostic purposes.^29^ In several studies, domain-specific knowledge was tested using standardized examination resources, such as an exam bank modeled after neurology board-style questions, 69 questions from the SCE Neurology Web Questions bank, and 120 neurologists’ 2022 neurology specialist exam results.^25^^,^^28^^,^^32^ Additionally, 20 neurophysiology questions focused on electrophysiological diagnosis were used.^30^ Furthermore, 30 challenging neuropathology cases combining morphological and genetic data for complex oncological diagnoses were examined.^31^ As part of some evaluations, neurosurgical interview questions were scored by neurosurgical consultants to assess reasoning in surgical settings.^27^ Moreover, some evaluations used a reinforcement learning framework to test adaptive performance using medical data as input.^26^ This collection of diverse datasets illustrates how LLMs in neurology require a wide range of skills, such as factual recall and multimodal diagnostic reasoning.

#### Model assessment

This section presents the significant potential of LLMs in transforming neuroscience research. Table 2 shows that ChatGPT-4 consistently exhibits high accuracy in different studies, suggesting its potential to aid in accurate diagnostics and research analysis.^25^^,^^28^^,^^29^^,^^32^ LLMs effectively diagnosed complex neuro-oncology cases, demonstrating their ability to handle intricate medical data.^31^ Two studies showed that LLMs are similar to human experts in certain tasks, suggesting that LLMs can assist human researchers by bringing additional insights and reducing workload.^24^^,^^27^ In a study by Cano-Besquet et al.,^29^ the authors pointed out that ChatGPT-4 can provide comprehensive and efficient analysis, which is vital for large-scale data processing in neuroscience, even though they do not always surpass human consultants in diagnostic accuracy. Another study revealed that models such as Microsoft Bing Chat can improve efficiency and accuracy significantly, indicating ongoing advancements in LLM capabilities.^26^ Studies by Shojaee-Mend et al.^30^ and Hewitt et al.^31^ show that the similar performance of these models across different languages and tasks underscores their versatility and adaptability in diverse research contexts.

**Table 2:** Summary of the best performing models.

Author	Place of study	**LLM Models**	**Best Model**	Comments
Kanzawa et al., 2024	Japan	Fine-tuned BERT	No clear winner	Similar performance to radiologists
Schubert, Wick, and Venkataramani 2023a	Germany	ChatGPT versions 3.5 (LLM 1) and 4 (LLM 2)	ChatGPT4	∗
Chen et al., 2023	USA	ChatGPT-powered bing chat	Microsoft bing chat	Potential improvement
Williams et al., 2024	UK	ChatGPT 3.5	No clear winner	Single model choice
Giannos 2023	UK	ChatGPT 3.5 legacy, ChatGPT3.5 default & ChatGPT4	ChatGPT4	∗
Cano-Besquet et al., 2024	USA	ChatGPT-4	ChatGPT4	∗
Shojaee-Mend et al., 2024	Iran	ChatGPT, Google's bard, and Anthropic's claude	No clear winner	Similar performance
Hewitt et al., 2024a	Germany	ChatGPT-4o, claude-3.5-sonnet, and Llama3	No clear winner	All effective in diagnosis
Ros-Arlanzón and Perez-Sempere 2024	Spain	ChatGPT3.5 and 4	ChatGPT4	∗

## Discussion

Recent advancements in LLM-based neurological research are reviewed and the potential of these models and their practical applications in this area are highlighted. The potential of LLMs in analyzing large medical records is being examined to aid in early diagnosis and support clinicians in neurology.^33^ Patient care and treatment planning for neurological disorders can be assisted by LLMs, but ethical and technical challenges such as data privacy and bias must be addressed.^34^^,^^35^ Neuroscience research has been transformed by recent advancements in LLMs. Enhancing data analysis, prediction, and application capabilities with self-supervised and transfer learning mean these models are poised to reshape the field.

### Incorporation of LLMs into neurological assessments

LLMs have shown proficiency in processing and synthesizing significant amounts of scientific literature, surpassing human experts in predicting the outcomes of neuroscience research. Neuroscience literature has refined models such as Brain GPT to improve prediction accuracy, demonstrating this capability.^36^ The potential of LLMs in analyzing large datasets from medical records, aiding in early diagnosis, and supporting clinicians in neurology is currently being investigated. An investigation was carried out to determine the effectiveness of well-tuned language models in classifying brain MRI reports into three categories, namely pretreatment, posttreatment, and nontumor cases, using data from 1207 MRI brain images.^24^ After being trained and tested, the customized BERT was found to be highly accurate and sensitive. The task was completed much faster than the radiologists, although it performed similarly. This paper's findings validate LLM's performance as comparable to human readers, with no significant differences in accuracy, with a 20 to 26 times faster processing speed. In,^25^ the authors stated that ChatGPT 4 performed better than the average human score when answering neurology board-style questions.

LLMs have demonstrated their proficiency in parsing and processing complex medical text, but their usefulness in neuro-oncology had not been thoroughly evaluated before this study. By integrating LLMs with Retrieval-Augmented Generation (RAG), neuropathology can benefit from valuable diagnostic support tools that assist in the interpretation and classification of complex conditions.^37^^,^^38^ It is reported that LLMs can aid clinical specialists in keeping up with evolving medical classifications.^31^ A similar trend can be observed in the other two works^28^^,^^32^ in answering questions about neurological-related board examinations. These studies pointed out that LLMs, particularly ChatGPT4, have been further refined and could have significant implications in clinical neurology.

ChatGPT4 model is a valuable diagnostic tool in inpatient neurology and can provide comprehensive and accurate initial diagnoses comparable to those made by consultant neurologists.^29^ Integrating models' initial diagnoses with those from consultants is suggested to achieve comprehensive diagnostics in all cases. Clinical decision-making could benefit from ChatGPT's ability to effectively evaluate neurological assessments, even though it occasionally produced hallucinatory scores when information was scarce. The ChatGPT-powered Bing Chat model demonstrated its ability to evaluate neurological assessments effectively, demonstrating potential in clinical decision-making, but occasionally resulted in hallucinated scores due to insufficient information being available.^26^ It appears that all of these are aimed at revolutionizing neuroscience research by improving data processing and predictive capabilities.

### Challenges and future perspectives

The revolutionizing influence of LLMs in neuroscience research is due to their enhanced data processing and predictive capabilities, but it is crucial to address ethical and technical challenges to fully realize their potential in this area. Privacy, data security, and potential biases in training data are concerns raised by the use of LLMs in neuroscience and it is essential to make sure that these models are applied safely and responsibly.^39^ The results of some of the research papers in this review indicated that while LLMs can perform better than human experts in certain predictive tasks, their integration with human expertise is necessary for making comprehensive scientific discoveries in neuroscience.^24^^,^^27^^,^^29^ For example, LLM showed a decreased performance in posttreatment brain tumor reports, which could limit its effectiveness in certain situations.^24^ It is not recommended to suggest that LLM can entirely replace doctors in these cases, as human expertise is still essential, especially for complex cases.

A study has been carried out to provide valuable information about the capabilities and limitations of LLMs in the field of neurophysiology.^30^ Although LLMs excel in general questions, they encounter difficulties with advanced reasoning and knowledge integration. Continuous, domain-specific assessments are essential for the evaluation and improvement of LLM performance. ChatGPT3.5 and 4 were identified as optimized models for lower-order questions in neurology-based questionnaires, which suggests the need to refine LLMs further.^25^ Another study showed that ChatGPT had an average error rate of 20 % across different test cases, which suggests that accuracy is hindered, particularly with complex wording or omitted details.^26^ AI and healthcare professionals could benefit from ChatGPT's potential to enhance neuroscience research, but there is still room for improvement to match human performance.^27^^,^^28^

ChatGPT4's improved performance highlights the potential of LLMs in specialized neurology education and practice.^25^ It is necessary to conduct more research to verify these findings and evaluate the influence of LLMs on neuroscience. However, ongoing development and collaboration between AI developers and medical experts are necessary to ensure the models' relevance and reliability in the evolving medical field. This topic is greatly supported by a study that mentions that integrating ChatGPT4's initial diagnoses with those from consultants could achieve comprehensive diagnostics in all cases.^29^ To effectively utilize the potential benefits of LLMs, it is recommended to implement them carefully and in a patient-centered way, especially with the advancement of technology to handle more complex neuroscience tasks.

### Limitations

The potential of LLMs in different domains of neurology is highlighted in this study, but it is crucial to discuss several limitations. Understanding the bias in data is crucial when using LLMs in neurological studies.^40^^,^^41^ The results and interpretations of the research can be affected by biases that are present in large datasets. Even though LLMs excel at specific tasks, they may not have the ability to generalize across different contexts and applications in neuroscience. Trying to understand how LLMs come to their conclusions can be challenging, which makes it challenging to interpret the results in a meaningful manner for neuroscience research.^40^^,^^42^ LLM training and deployment can be a limitation for many research institutions due to the significant computational resources required. Ethical considerations regarding data privacy and the potential misuse of AI-generated insights are raised using LLMs in sensitive areas such as neuroscience.

## Conclusions

This study outlines recent advances in LLM chatbots and how they can transform multiple domains of neurology, from diagnostics to education. Our study is the first to compare LLM chatbot applications across such diverse neurological contexts, including neuroimaging interpretation, clinical diagnostics, neurophysiology, neuropathology, and preparing for specialist exams. However, we found challenges in ensuring data privacy, interpretability, and domain-specific fine-tuning, despite LLMs' ability to improve workflows, decision support, and access. A heavy reliance on AI-generated outputs without adequate human oversight may compromise clinical safety, which emphasizes the importance of a hybrid human-AI collaboration. To ensure reliability, fairness, and clinical relevance, future research should prioritize developing neurology-specific LLMs trained on curated datasets. With these challenges addressed, LLM chatbots could foster interdisciplinary collaboration, elevate patient care, and advance both research and education.

## Authors contributions

All authors contributed equally. All authors have critically reviewed and approved the final draft and are responsible for the content and similarity index of the manuscript.

## Ethical approval

Ethical approval was not required for this study.

## Source of funding

This work did not receive funds from other sources.

## Conflicts of interest

The authors have no conflicts of interest to declare.
